# The Price of a Neglected Zoonosis: Case-Control Study to Estimate Healthcare Utilization Costs of Human Brucellosis

**DOI:** 10.1371/journal.pone.0145086

**Published:** 2015-12-15

**Authors:** Oded Vered, Tzahit Simon-Tuval, Pablo Yagupsky, Miki Malul, Assi Cicurel, Nadav Davidovitch

**Affiliations:** 1 Department of Public Policy & Administration, Guilford Glazer Faculty of Business and Management, Ben-Gurion University of the Negev, Beer-Sheva, Israel; 2 Department of Health Systems Management, Guilford Glazer Faculty of Business and Management and Faculty of Health Sciences, Ben-Gurion University of the Negev, Beer-Sheva, Israel; 3 Clinical Microbiology Laboratory, Soroka University Medical Center, Ben-Gurion University of the Negev, Beer-Sheva, Israel; 4 Clalit Health Services, Southern District and Faculty of Health Sciences, Ben-Gurion University of the Negev, Beer-Sheva, Israel; Institut National de la Recherche Agronomique, FRANCE

## Abstract

Human brucellosis has reemerged as a serious public health threat to the Bedouin population of southern Israel in recent years. Little is known about its economic implications derived from elevated healthcare utilization (HCU). Our objective was to estimate the HCU costs associated with human brucellosis from the insurer perspective. A case-control retrospective study was conducted among Clalit Health Services (CHS) enrollees. Brucellosis cases were defined as individuals that were diagnosed with brucellosis at the Clinical Microbiology Laboratory of Soroka University Medical Center in the 2010–2012 period (n = 470). Control subjects were randomly selected and matched 1:3 by age, sex, clinic, and primary physician (n = 1,410). HCU data, demographic characteristics and comorbidities were obtained from CHS computerized database. Mean±SD age of the brucellosis cases was 26.6±17.6 years. 63% were male and 85% were Bedouins. No significant difference in Charlson comorbidity index was found between brucellosis cases and controls (0.41 *vs*. 0.45, respectively, *P = 0*.*391*). Before diagnosis (baseline), the average total annual HCU cost of brucellosis cases was slightly yet significantly higher than that of the control group ($439 *vs*. $382, *P<0*.*05*), however, no significant differences were found at baseline in the predominant components of HCU, i.e. hospitalizations, diagnostic procedures, and medications. At the year following diagnosis, the average total annual HCU costs of brucellosis cases was significantly higher than that of controls ($1,327 *vs*. $380, respectively, *P<0*.*001*). Most of the difference stems from 7.9 times higher hospitalization costs (*p<0*.*001*). Additional elevated costs were 3.6 times higher laboratory tests (*P<0*.*001*), 2.8 times higher emergency room visits (*P<0*.*001*), 1.8 times higher medication (*P<0*.*001*) and 1.3 times higher diagnostic procedures (*P<0*.*001*). We conclude that human brucellosis is associated with elevated HCU costs. Considering these results in cost-effective analyses may be crucial for both reducing health inequities and optimal allocation of health systems’ scarce resources.

## Introduction

Human brucellosis is a common zoonotic disease, yet its incidence varies between as well as within countries [1, 2]. It is mostly prevalent in low and middle-income countries in the Mediterranean basin, the Middle East, and central Asian countries [3], however it still remains an important public health concern in developed countries as well [2, 3]. The prevalence rate of human brucellosis is associated with socioeconomic factors, the effectiveness of the surveillance system, the presence of livestock brucellosis control programs, and international tourism [3, 4]. Human brucellosis is acquired through direct contact with infected animals, placentas, aborted fetuses, or through consumption of unpasteurized dairy products [2, 3]. In Israel, human brucellosis is prevalent mostly among Bedouins. Although the Bedouin society is gradually moving from seminomadic lifestyle to permanent settlements and modern agriculture, still relatively high rates of unvaccinated sheep and goats and consumption of unpasteurized dairy products create high risk for brucellosis infection [5]. According to cases reported to the Ministry of Health the national incidence rate per 100,000 population in 2009 was 7.0 among Arabs [5], however a more serious public health problem has emerged in the southern district where a minimal incidence rate of 50.2 per 100,000 residents, and 151.9 per 100,000 Bedouin individuals were observed in 2012 [6].

The initial clinical manifestations of human brucellosis include fever, sweat, fatigue, headache and joint pain that can last for weeks to months [7]. The disease may progress to debilitating symptoms such as arthralgia, myalgia and back pain. In addition the disease may be associated with hepatomegaly, splenomegaly, overt arthritis, spondylitis, respiratory diseases, or epididymo-orchitis mimicking other infectious and non-infectious conditions. Less prevalent but more severe and even fatal presentations include endocarditis and neuropsychiatric complications. Due to the nonspecific features of human brucellosis, the diagnosis is frequently not entertained when patients present at the healthcare facilities [3]. Because of the protean clinical manifestations of the disease and the need for prolonged and specific combination therapy with antibiotics that are not used in other infectious, human brucellosis should be confirmed by adequate laboratory means including culture, serology, and nucleic-acid amplification assays [8]. The preferred treatment in human brucellosis without complications is doxycycline-aminoglycoside combination, however alternative oral treatments are still to be considered, specifically if parental administration of aminoglycoside is not feasible and healthcare personnel is limited [3, 9, 10].

Human brucellosis prevention and control strategies are targeted at reducing animal-to-human transmission and include livestock vaccination, test and slaughter of infected animals, and education of populations living in endemic regions for the disease to avoid unpasteurized dairy foods, cook meat thoroughly, and use precautions in high-risk occupations such as for farmers, veterinarians or laboratory workers [11–14]. Due to budget constraints and competing interests, in order to justify allocation of resources to control the disease, it is essential to provide public health policy-makers economic evaluation of the alternative strategies. Similarly to other zoonoses, this economic evaluation requires integrating the agricultural and human aspects of the disease [15]. The economic impact on the agricultural sector refers to the direct cost of the control strategies as well as the decrease in livestock production. The economic impact from the human health perspective refers to the cost of diagnosis and treatment and the cost of earnings and productivity loss due to impaired functioning and absenteeism [16–18].

The empirical research evaluating the diagnosis and treatment costs of the disease, i.e. healthcare utilization (HCU) of patients with human brucellosis is limited. Most of the evidence was based on Delphi expert opinion and population surveys [16, 19]. To the best of our knowledge, a single study examined the actual costs of hospitalizations before and after the implementation of brucellosis control program, yet only on an aggregate level [17]. The purpose of the current study was to examine annual HCU of patients with brucellosis before and after diagnosis and compared to healthy controls from the insurer perspective. Our single-payer setting enabled us to provide reliable and comprehensive measures of HCU data for a representative cohort of patients. The implementation of effective brucellosis control intervention necessitates comprehensive analysis of the burden of disease that is based on accurate and objective measures as provided in the current study.

## Materials and Methods

### Subjects

A case-control retrospective study was conducted among members of Clalit Health Services (CHS), the largest health maintenance organization (HMO) in Israel.

Brucellosis cases were defined as individuals that were diagnosed with brucellosis by positive blood culture and/or positive serology test (positive Rose-Bengal test followed by a standard agglutination test (SAT) titer ≥160), at the Clinical Microbiology Laboratory of Soroka University Medical Center (CML-SUMC) in the 2010–2012 period (n = 614).

Control subjects were CHS enrollees who were not diagnosed with brucellosis in the same period, and were randomly selected and matched 1:3 to the brucellosis cases by age, gender, clinic, and primary care physician (control group A). One hundred forty-four brucellosis cases were excluded from analyses due to inconsistent eligibility in the follow-up period and/or lack of matched control subjects. Hence, the final study population consisted of 470 brucellosis cases and 1410 controls. No significant differences were found between the final brucellosis cases that were included in the analyses (n = 470) and those who were excluded (n = 144) with regard to characteristics that are known to affect HCU, namely age (*P = 0*.*060*), gender (*P = 0*.*659*), % of Bedouins (*P = 0*.*152*), socioeconomic status (SES) (*P = 0*.*068*) and Charlson comorbidity index (CCI) (*P = 0*.*112*). Thus, the potential selection bias is minimized. The study was approved by Meir Medical Center Review Board- Helsinki Committee and SUMC Review Board- Helsinki Committee (#057/2013 and #0108-13SOR, respectively). Following these committees’ recommendation, written informed consent of study population was not required and patients’ information was anonymized and de-identified prior to analysis. Matching brucellosis cases to the control group A was designed to ensure similar patterns of HCU, but could lead to biases due to close family ties and related place of residency that characterizes the Bedouin society. These may increase the odds that undiagnosed human brucellosis could be prevalent among control subjects as well. In order to minimize this bias, brucellosis cases (n = 463) were matched to additional control subjects (control group B, n = 1389) who were not residents of the same neighborhoods and/or villages as those of the brucellosis cases. This control group was matched 1:3 to brucellosis cases by age, gender and ethnicity (Jewish/Bedouin).

### Healthcare utilization

The month of diagnosis was defined as the index date. Information regarding HCU was obtained from CHS computerized medical databases for one year before the index date and one year following it (data for subjects in the control groups were extracted for a follow-up period identical to that of their matched brucellosis cases). HCU included: hospitalizations; diagnostic procedures including CT scans, ultrasounds and MRIs; medications (according to the WHO anatomical therapeutic chemical (ATC) classification system [20]; surgical procedures such as cardiac catheterization and heart or spinal column; visits to specialists; emergency room visits; outpatients visits; and laboratory tests. All costs were adjusted to 2013 prices and converted into USD ($) using an exchange rate of 3.6 Israeli Shekels per USD.

### Additional measures

Additional measure included: demographic characteristics (age, gender, percentage of Bedouins); SES defined as: low (1), average (2), high (3) according to the primary care clinic address, and CCI [21].

### Statistical analysis

Comparison between study and control groups’ age, SES, CCI and HCU (that were not normally distributed) was done using the Mann-Whitney U test. Comparison between groups’ proportions (e.g. gender) was done using Chi-square test. Comparison of annual HCU between years within each group was done using the Wilcoxon matched-pairs signed-ranks test. Data were analyzed using STATA software (version 11.0, StataCorp, College Station, TX, USA). *P* values *<0*.*05* determined statistical significance in all analyses.

## Results

Four hundred seventy brucellosis cases were included in the analysis (median age of 21.0 years, 62.6% male and 84.9% Bedouins). As expected no significant differences were found between brucellosis cases and control group A with regard to age, gender, percentage of Bedouins and SES (Table 1). In addition, no significant difference was found between groups with regard to CCI and both groups demonstrated low comorbidity burden (0.41 *vs*. 0.45, respectively, *P = 0*.*391*).

**Table 1:** Characteristics of study population.

	Brucellosis cases	Control group A	*P Value*
n	470	1410	-
% Male	62.6%	62.6%	1.000^a^
% Bedouins	84.9%	81.9%	.140^a^
Age^b^	26.62 ± 17.64 (21, 1–85)	26.64 ± 17.66 (21, 1–86)	0.991ᶜ
SES^b^,^d^	1.01 ± 0.09 (1, 1–2)	1.01 ± 0.09 (1, 1–2)	1.000ᶜ
CCI^b^	0.41 ± 1.12 (0, 0–7)	0.45 ± 1.15 (0, 0–9)	0.391ᶜ

Abbreviations: SES- Socioeconomic status; CCI- Charlson comorbidity index.

^a^ Chi-square test.

^b^ Values are mean ± SD (median, min-max).

^c^ Mann-Whitney U test.

^d^ SES was defined as: low (1), average (2), high (3) according to the enrollee's community clinic.

As presented in Table 2, at the year before the diagnosis was established (baseline), The average annual total HCU cost of brucellosis cases was slightly yet significantly higher than that of the control group ($439 *vs*. $382, *P<0*.*05*). However, no significant differences were found at baseline in the predominant components of HCU, specifically, in hospitalizations cost ($141 *vs*. $108, *P>0*.*05*), diagnostic procedures cost ($43 *vs*. $44, *P>0*.*05*), medications cost ($58 *vs*. $63, *P>0*.*05*), and surgical procedures ($86 *vs*. $73, *P>0*.*05*). Significant difference was found at baseline in emergency room visits costs ($46 *vs*. $34, *P<0*.*05*).

**Table 2:** Annualized healthcare utilization before and after diagnosis, by study group.

	Before diagnosis		After diagnosis	
	Brucellosis cases (n = 467)	Control group A (n = 1404)	Brucellosis cases (n = 467)	Control group A (n = 1404)
**Total Cost** Costs (2013USD)	439 ± 1592 (52, 6–260)	382 ± 1495 (36, 3–209)*	1327 ± 2347 (320, 142–1349)^##^	380 ± 1472 (40, 2–231)**
**Hospitalization** Costs (2013USD)	141± 893 (0,0–0)	108 ± 716 (0,0–0)	892 ± 2053 (0, 0–636) ^##^	113 ± 799 (0, 0–0) **
Number of hospitalization	0.10 ± 0.54 (0, 0–0)	0.08 ± 0.45 (0, 0–0)	0.52 ± 1.17 (0, 0–1) ^##^	0.08 ± 0.54 (0, 0–0) **
Number of days	0.23 ± 1.44 (0, 0–0)	0.20 ± 1.28 (0, 0–0)	1.67 ± 3.79 (0, 0–1) ^##^	0.20 ± 1.41 (0, 0–0) **
% Hospitalized	6.0	5.0	27.6	5.0 ^ʃ^
Length of stay	3.89 ± 4.59 (2, 1–5)	3.91 ± 4.33 (3, 2–4)	6.03 ± 5.08 (4, 3–7)	4.09 ± 4.91 (3, 1–5) **
**Long-term hospitalization** Costs (2013USD)	11 ± 236 (0, 0–0)	4 ± 152 (0, 0–0)	12 ± 262 (0, 0–0)	3 ± 114 (0, 0–0)
Number of hospitalization	0.01 ± 0.19 (0, 0–0)	0.00 ± 0.08 (0, 0–0)	0.01 ± 0.19 (0, 0–0)	0.00 ± 0.05 (0, 0–0)
Number of days	0.06 ± 1.34 (0, 0–0)	0.02 ± 0.83 (0, 0–0)	0.07 ± 1.43 (0, 0–0)	0.02 ± 0.64 (0, 0–0)
**Diagnostic procedures** Costs (2013USD)	43 ± 114 (0, 0–34)	44 ± 143 (0, 0–22)	62 ± 158 (0, 0–50)^##^	46 ± 145 (0, 0–26)**
Number of procedures	1.12 ± 2.17 (0, 0–2)	0.97 ± 2.12 (0, 0–1)*	1.41 ± 2.13 (0, 0–2)^##^	0.96 ± 1.96 (0, 0–1) **
**Medications** Costs (2013USD)	58 ± 436 (11, 2–30)	63 ± 410 (8, 0–27)	108 ± 540 (44, 27–70)^##^	59 ± 310 (8, 0–30) **
Number of Rx	9.36 ± 14.15 (4, 1–12)	9.95 ± 18.39 (3, 1–10) *	14.54 ± 15.35 (10, 6–17)^##^	10.12 ± 18.93 (3, 0–10) **
**Surgical procedures** Costs (2013USD)	86 ± 727 (0, 0–0)	73 ± 824 (0, 0–0)	30 ± 360 (0, 0–0)	59 ± 641 (0, 0–0)
Number of surgeries	0.02 ± 0.15 (0, 0–0)	0.01 ± 0.12 (0, 0–0)	0.01 ± 0.10 (0, 0–0)	0.02 ± 0.16 (0, 0–0)
**Specialist visits** Costs (2013USD)	31 ± 65 (0, 0–35)	29 ± 66 (0, 0–31)	37 ± 65 (0, 0–49)^#^	30 ± 65 (0, 0–30) *
Number of visits	1.37 ± 2.69 (0, 0–2)	1.28 ± 2.73 (0, 0–2)	1.58 ± 2.66 (0, 0–2)^#^	1.33 ± 2.84 (0, 0–2) *
**Emergency Room visits** Costs (2013USD)	46 ± 103 (0, 0–0)	34 ± 98 (0, 0–0)*	118 ± 175 (0, 0–179) ^##^	42 ± 105 (0, 0–0) **
Number of visits	0.26 ± 0.58 (0, 0–0)	0.19 ± 0.55 (0, 0–0) *	0.67 ± 0.99 (0, 0–1) ^##^	0.23 ± 0.58 (0, 0–0) **
**Outpatient visits** Costs (2013USD)	9 ± 84 (0, 0–0)	10 ± 187 (0, 0–0)	11 ± 133 (0, 0–0)	12 ± 198 (0, 0–0)
Number of visits	0.03 ± 0.27 (0, 0–0)	0.03 ± 0.52 (0, 0–0)	0.03 ± 0.46 (0, 0–0)	0.03 ± 0.51 (0, 0–0)
**Laboratory tests** Costs (2013USD)	14 ± 32 (1, 0–18)	16 ± 78 (0, 0–14) *	58 ± 56 (46, 27–74) ^##^	16 ± 112 (0, 0–13) **
Number of tests	8.54 ± 12.90 (1, 0–15)	7.34 ± 13.11 (0, 0–14) *	21.40 ± 19.35 (17, 6–31) ^##^	7.94 ± 14.99 (0, 0–14) **

Values are mean ±SD (median, 25 percentile-75 percentile).

* Mann-Whitney U test, *P<0*.*05*;

** *P<0*.*001*.

^#^ Wilcoxon matched-pairs signed-ranks test, *P<0*.*05*;

^##^ *<0*.*001*.

^ʃ^ Chi-squared test, *P<0*.*001*.

At the year following diagnosis, the average total annual HCU costs of brucellosis cases was significantly higher than the control group ($1,327 *vs*. $380, respectively, *P<0*.*001*). Most of the difference stems from 7.9 times higher hospitalization costs ($892 *vs*. $113, respectively *P<0*.*001*). Moreover, compared to control group significantly higher percentage of brucellosis cases were hospitalized (27.6% *vs*. 5.0%, *P<0*.*001*) and their average length of stay was higher (6.03 days *vs*. 4.09 days, *P<0*.*001*). Additional elevated utilization costs were 3.6 times higher laboratory tests (*P<0*.*001*), 2.8 times higher emergency room visits (*P<0*.*001*), 1.8 times higher medication usage (*P<0*.*001*) and 1.3 times higher diagnostic procedures (*P<0*.*001*).

The average annual total HCU costs of brucellosis cases following diagnosis was 3.0 times higher compared to the year before diagnosis was established ($1,327 *vs*. $439, *P<0*.*001*). Significant differences were found in hospitalizations cost ($892 *vs*. $141, *P<0*.*001*), emergency room visits costs ($118 *vs*. $46, *P<0*.*001*), medications costs ($108 *vs*. $58, *P<0*.*001*), diagnostic procedures costs ($62 *vs*. $43, *P<0*.*001*) and laboratory tests costs ($58 *vs*. $14, *P<0*.*001*). This trend was not observed with regard to long-term hospitalizations, surgical procedures, and outpatient visits costs (Table 2).

Table 3 presents comparisons of utilization of diagnostic procedures that are possibly related to human brucellosis. Although no significant difference in total diagnostic procedures costs was found between groups at baseline, a significant difference was found at baseline in spinal/skeletal diagnosis costs ($7 *vs*. $5, *P<0*.*05*). These costs increased significantly among brucellosis cases following diagnosis ($10 *vs*. $7, *P<0*.*05*). In addition, no differences were found between groups in utilization of MRI at baseline, yet compared to the year before diagnosis, significant increase in MRI costs ($12 *vs*. $1, *P<0*.*05*) was observed among brucellosis cases. No significant differences in utilization of CT scans were observed both between groups and between years of follow-up (Table 3).

**Table 3:** Annualized utilization of diagnostic procedures before and after diagnosis, by study group.

	Before diagnosis		After diagnosis	
	Brucellosis cases (n = 467)	Control group A (n = 1404)	Brucellosis cases (n = 467)	Control group A (n = 1404)
**Total diagnostic procedures** Costs (2013USD)	43± 114 (0, 0–34)	44± 143 (0, 0–22)	62± 158 (0, 0–50)##	46± 145 (0, 0–26)**
Number of visits	1.12± 2.17 (0, 0–2)	0.97± 2.12 (0, 0–1)*	1.41± 2.13 (0, 0–2)##	0.96± 1.96 (0, 0–1)**
**Spinal/Skeletal** Costs (2013USD)	7± 29 (0, 0–0)	5± 20 (0, 0–0)*	10± 29 (0, 0–0)#	4± 20 (0, 0–0)**
Number of procedures	0.34± 1.15 (0, 0–0)	0.24± 0.82 (0, 0–0)*	0.42± 1.00 (0, 0–0)#	0.21± 0.79 (0, 0–0)**
**MRI** Costs (2013USD)	1± 24 (0, 0–0)	4± 54 (0, 0–0)	12± 104 (0, 0–0)#	7± 75 (0, 0–0)
Number of procedures	0.00± 0.05 (0, 0–0)	0.01± 0.10 (0, 0–0)	0.02± 0.17 (0, 0–0)#	0.01± 0.13 (0, 0–0)
**CT** Costs (2013USD)	6± 27 (0, 0–0)	4± 25 (0, 0–0)	5± 24 (0, 0–0)	4±23 (0, 0–0)
Number of procedures	0.05± 0.26 (0, 0–0)	0.04± 0.21 (0, 0–0)	0.05± 0.23 (0, 0–0)	0.04± 0.20 (0, 0–0)

Values are mean ±SD (median, 25 percentile-75 percentile).

* Mann-Whitney U test, *P<0*.*05*;

** *P<0*.*001*.

^#^ Wilcoxon matched-pairs signed-ranks test, *P<0*.*05*;

^##^ *<0*.*001*.

Table 4 presents comparisons of utilization of brucellosis-related treatment medications. Significant differences between groups were found at baseline in aminoglycoside antibacterials (J01G) costs (*P<0*.*001*) and this utilization has increased significantly following diagnosis among brucellosis cases ($22 *vs*. $1, *P<0*.*001*). It should be noted that unlike oral medications, utilization of aminoglycosides (J01G) is associated with additional costs (nursing staff and equipment) derived from the need for parenteral administration, and these costs were not included in our analyses. Significant increase in brucellosis-related medication following diagnosis among brucellosis cases was also observed in analgesics (N02), as well as tetracyclines (J01A), sulfonamides and trimethoprim (J01E) and rifampicin (J04A) which are also employed in the treatment of human brucellosis (Table 4).

**Table 4:** Annualized utilization of medications before and after diagnosis, by study group.

	Before diagnosis		After diagnosis	
	Brucellosis cases (n = 467)	Control group A (n = 1404)	Brucellosis cases (n = 467)	Control group A (n = 1404)
**Total medication** Costs (2013USD)	58± 436 (11, 2–30)	63± 411 (8, 0–27)	108± 540 (44, 27–70)#	59± 310 (8, 0–30)**
Number of Rx.	9.36± 14.15 (4, 1–12)	9.95± 18.39 (3, 1–10)*	14.54± 15.35 (10, 6–17)#	10.12± 18.93 (3, 0–10)**
**Analgesics (N02)** Costs (2013USD)	1± 3 (0, 0–1)	2± 20 (0, 0–1)	2± 8 (0, 0–2)#	2± 17 (0, 0–1)**
Number of Rx.	0.99± 1.99 (0, 0–1)	1.08± 2.74 (0, 0–1)	1.37± 2.01 (1, 0–2)#	0.97± 2.36 (0, 0–1)**
**Tetracyclines (J01A)** Costs (2013USD)	0± 1 (0, 0–0)	0± 0 (0, 0–0)**	3± 2 (3, 1–4)#	0 ± 1 (0, 0–0)**
Number of Rx.	0.10± 0.44 (0, 0–0)	0.03± 0.18 (0, 0–0)**	1.73± 1.32 (2, 1–2)#	0.04± 0.24 (0, 0–0)**
**Aminoglycoside antibacterials (J01G)** Costs (2013USD)	1± 4 (0, 0–0)	0± 1 (0, 0–0)**	22± 16 (17, 13–32)#	0± 0 (0, 0–0)**
Number of Rx.	0.07± 0.44 (0, 0–0)	0.00± 0.04 (0, 0–0)**	1.84± 1.74 (1, 1–2)#	0.00± 0.05 (0, 0–0)**
**Sulfonamides and Trimethoprim (J01E)** Costs (2013USD)	0± 1 (0, 0–0)	0± 0 (0, 0–0)	1± 3 (0, 0–0)#	0± 1 (0, 0–0)**
Number of Rx.	0.02± 0.27 (0, 0–0)	0.01± 0.25 (0, 0–0)	0.27± 0.91 (0, 0–0)#	0.02± 0.34 (0, 0–0)**
**Quinolone antibacterials (J01M)** Costs (2013USD)	0± 1 (0, 0–0)	0± 0 (0, 0–0)	0± 1 (0, 0–0)	0± 1 (0, 0–0)*
Number of Rx.	0.08± 0.32 (0, 0–0)	0.06± 0.30 (0, 0–0)	0.11± 0.55 (0, 0–0)	0.06± 0.43 (0, 0–0)*
**Drugs for treatment of tuberculosis (J04A)** Costs (2013USD)	0± 0 (0, 0–0)	0± 0 (0, 0–0)	2± 11 (0, 0–0)#	0± 3 (0, 0–0)**
Number of Rx.	0± 0 (0, 0–0)	0± 0 (0, 0–0)	0.08± 0.38 (0, 0–0)#	0.00± 0.11 (0, 0–0)**
**Antibiotics for topical use (D06A)** Costs (2013USD)	0± 1 (0, 0–0)	0± 1 (0, 0–0)	0± 5 (0, 0–0)	0± 3 (0, 0–0)
Number of Rx.	0.12± 0.38 (0, 0–0)	0.10± 0.32 (0, 0–0)	0.09± 0.33 (0, 0–0)	0.08± 0.33 (0, 0–0)

Values are mean ±SD (median, 25 percentile-75 percentile).

* Mann-Whitney U test, *P<0*.*05*;

** *P<0*.*001*.

^#^ Wilcoxon matched-pairs signed-ranks test, *P<0*.*001*.

Comparisons between brucellosis cases (n = 460) and control group B (n = 1388) with regard to all components of HCU yielded similar trends. At the year following diagnosis, the average total annual HCU costs of brucellosis cases was significantly higher than the control group ($1,347 *vs*. $372, respectively, *P<0*.*001*). Most of the difference stems from 7.1 times higher hospitalization costs ($890 *vs*. $125, respectively *P<0*.*001*). Additional elevated utilization costs were 4.7 times higher laboratory tests ($59 *vs*. $12, respectively, *P<0*.*001*), 2.7 times higher emergency room visits ($120 *vs*. $44, respectively, *P<0*.*001*), 1.8 times higher medication usage ($112 *vs*. $61, respectively, *P<0*.*001*) and 1.5 times higher diagnostic procedures ($63 *vs*. $41, respectively, *P<0*.*001*).

## Discussion

Our analyses reveal that human brucellosis is associated with elevated HCU that stem predominantly from higher hospitalization costs. In addition, compared to control subjects, patients with human brucellosis had higher utilization of medications, spinal/skeletal diagnostic procedures, emergency room visits, and laboratory tests. The following discussion considers these results in light of the currently available literature.

Human brucellosis was associated with 7.9 times higher hospitalization costs compared to controls. Similar trend was observed in a study conducted in Portugal [17] where hospitalization costs had decreased considerably following a five-year brucellosis control program. This higher utilization may be derived from three reasons: First, brucellosis is one of the “great imitators” and patients with the disease frequently present with symptoms and signs mimicking other infectious and non-infectious conditions such as rheumatic diseases, hepatitis, hematological disorders, etc. In addition, although the disease has low mortality rate and usually respond to adequate antibiotic therapy, in some patients development of focal complications such as sacroiliitis or abscess formation, require additional work-up which may include MRI studies or surgical procedures. Thus, even after the diagnosis of brucellosis is confirmed, patients may be hospitalized for further investigation and a wide array of laboratory tests and costly imaging studies are performed. Second, in some patients long hospitalization may be required because of the need for parenteral aminoglycoside administration which not always can be given at the community setting. Finally, the clinical course of human brucellosis is characterized by frequent relapses which may occur shortly after completion of antibiotic treatment, thus requiring repeat utilization of medical services. Similar to our results, a targeted group surveys in Turkey found that 28% of patients are hospitalized [19], however a household survey from Mongolia estimated that 50% of diagnosed patients are hospitalized [16]. In addition, while these surveys revealed that the average length of stay is 11 days [19] or 21 days [16], our analyses of objective computerized data suggest that the average length of stay was 6 days in the year following diagnosis. The fact that hospitalization is a core component of HCU, and highly affects benefit-cost evaluation of eradication strategies [16], emphasizes the importance of analysis of actual (rather than self-reported) HCU data as observed in the current study.

Delphi expert opinion surveys suggested that only 35% of brucellosis patients that seek medical attention for the first time are correctly diagnosed due to unspecific symptoms [19]. A study from northern Tanzania [22] revealed that while malaria was uncommon and over-diagnosed among patients hospitalized for febrile illness, human brucellosis (amongst other acute bacterial zoonoses) was common, yet frequently missed. A 12-months prospective study conducted among Bedouins patients hospitalized in southern Israel for febrile disease, found that 60% of patients with zoonotic infections (brucellosis amongst others) were misdiagnosed, and only 57% received adequate antibiotic treatment [23]. This diagnostic challenge may also be reflected in the elevated number of emergency room visits of brucellosis cases before diagnosis was established compared to the control group. These insights highlight the economic implications of delayed diagnosis and the need to increase public and physicians’ awareness to the wide spectrum of symptoms and non-specific presentation of the infection in humans. In addition, it may be required to encourage the use of practical diagnostic tests such as Rose-Bengal assay in the emergency room setting [24]. Targeting patients with human brucellosis effectively and in a timely manner may improve their health outcomes, preventing complications and progression to the difficult-to-treat chronic stage, decreasing hospital length of stay, or even avoiding hospitalizations altogether.

Our analyses revealed elevated utilization of spinal/skeletal diagnostic procedures among patients with brucellosis. This result correspond with a recent systematic review that assessed the frequency and severity of clinical manifestations of human brucellosis [7] and found that debilitating conditions such as arthralgia and back pain where prevalent among 65% and 45% of patients, respectively. Based on this insight it was assumed that the consumption of analgesics will be higher among brucellosis cases compared to the control group. However, although a significant difference was observed between groups, it was relatively marginal. It is worth noting that since most of disease burden occurs in less developed communities, where occupations commonly involve physical activities, the impact of musculoskeletal pain and dysfunction may be even worse than the sole aspect of elevated HCU. Further research should be focused on estimation of measures such as productivity loss and decreased earnings due to absenteeism that are associated with human brucellosis and were not addressed in the current study.

Our analysis has several limitations. First, our analysis relied on financial databases that lack clinical information such as disease severity or diagnosis at hospital discharge. Thus, we could not provide a thorough explanation for the elevated hospitalizations cost that was observed among patients who were diagnosed with human brucellosis and we could not stratify results to disease severity subgroups. Second, cost estimates of HCU may not be generalizable to other healthcare systems, as practice patterns and cost estimates may differ. This limitation does not weaken our analyses since our objective was to estimate the difference in HCU between groups rather than to refer to an absolute value. Third, naturally, the current analyses of HCU focused on patients with symptomatic disease. However asymptomatic human brucellosis exists [25], thus in order to achieve early diagnosis of human brucellosis and prevent its chronic manifestation, clinicians’ awareness to the asymptomatic infection should be improved. Finally, our short-term analyses provided insight with regard to HCU one year following diagnosis, however treatment failure or relapse are relatively frequent [3, 19], thus long-term analyses of HCU may be warranted.

Notwithstanding these limitations, to the best of our knowledge, our study is the first to provide a comprehensive description of all-cause HCU of patients diagnosed with human brucellosis. Our results reveal that human brucellosis is associated with elevated HCU costs. Further research focusing on disease recurrence rate will enable to examine whether eradication of brucellosis would be associated with long-term reduction in HCU. Exploring long-term patterns of HCU following diagnosis may provide complementary data for cost-effectiveness analysis. It was previously shown that whereas controlling brucellosis by livestock mass vaccination is not cost-effective from a sole public health perspective, it is highly cost-effective when costs are attributed to the health and the agricultural sectors in proportion to their benefits [16]. Following the “one health” framework for estimating the economic costs of zoonoses from a societal perspective [15], considering our results in cost-effective analyses may be crucial for both reducing health inequities and optimal allocation of health systems’ scarce resources.
